# Central nervous system relapse in triple-negative breast cancer patients achieving pathological complete response after neoadjuvant chemotherapy: A retrospective cohort analysis

**DOI:** 10.1016/j.breast.2025.104553

**Published:** 2025-08-05

**Authors:** Gabriel Berlingieri Polho, Yumi Ricucci Shinkado, Leticia Kimie Murazawa, Vinicius Vitor Oliveira, Victor Rocha Pinheiro, Diana del Cisne Pineda Labanda, Romualdo Barroso-Sousa, Luciana Rodrigues Carvalho Barros, Laura Testa, Renata Colombo Bonadio

**Affiliations:** a Instituto do Câncer do Estado de São Paulo, Brazil, Av. Dr. Arnaldo, 251 – Cerqueira Cesar, São Paulo, SP, Brazil; b Dasa Oncology, Brasilia Hospital, SHIS QI 15, Lago Sul, Brasília, Brazil; c Instituto D’Or de Pesquisa e Ensino (IDOR), Av. Brigadeiro Luís Antônio, 5001, Jardim Paulista, São Paulo, SP, Brazil

**Keywords:** Triple-negative, Breast cancer, Neoadjuvant, Recurrence

## Abstract

Triple-negative breast cancer (TNBC) is associated with an increased risk of distant metastases. While pathological complete response (pCR) after neoadjuvant chemotherapy predicts favorable outcomes, data suggest it may not uniformly protect against central nervous system (CNS) relapse.

This study analyzed 774 TNBC patients to assess recurrence patterns. While CNS recurrences were numerically lower in pCR patients (5.0 % vs 8.8 %, p = 0.11), they represented a significantly higher proportion of all relapses in this group (50.0 % vs 20.2 %, p = 0.003). These results suggest that while pCR reduces overall recurrence risk, there is a distinct pattern of CNS-predominant failure.

## Highlights

• CNS relapses remain an issue in TNBC patients, even after pCR.
• pCR lowered overall recurrence, but CNS accounted for 50 % of relapses.
• Non-pCR patients had more extracranial and locoregional recurrences.
• CNS risk may reflect tumor biology or sanctuary effects in pCR patients.
• Findings call for CNS-targeted follow-up in pCR TNBC populations.

## 1 Introduction

Triple-negative breast cancer (TNBC) is characterized by a high propensity for visceral metastases, including recurrence in the central nervous system (CNS). Pathological complete response (pCR) following neoadjuvant chemotherapy (NACT) is widely regarded as a favorable prognostic marker. However, recent reports challenge the assumption that pCR uniformly reduces recurrence risk across all metastatic sites.

In a post-hoc analysis of KN 522, while fewer pCR patients experienced recurrence overall, those who did relapse showed a disproportionate tendency for CNS involvement compared to non-pCR patients [1]. This observation suggests that pCR, while beneficial for systemic control, may not effectively prevent the risk of CNS progression. Although similar findings were presented in *post-hoc* analysis of SPY-2 trials [2] and in a retrospective cohort [3], literature on these findings are scarce.

In this study, we retrospectively analyzed a cohort of TNBC patients treated with NACT to evaluate the relationship between pCR status and patterns of recurrence, with a focus on CNS relapse.

## 2 Methods

This single-center, retrospective cohort study included female patients with stage II–III TNBC treated with NACT between 2013 and 2024. Patients with TNBC who received NACT were identified from an electronic database, after filtering the institutional patient registry. TNBC was defined as tumors lacking expression of estrogen receptor (ER), progesterone receptor (PR), and HER2 (by immunohistochemistry and/or fluorescence in situ hybridization). We excluded patients with metastatic disease at diagnosis, patients with synchronous bilateral breast cancer, or with second primary tumor (except localized non-melanoma skin cancer) and those with unavailable data on electronic medical records. Clinical data were extracted from electronic medical records, including baseline demographics, tumor characteristics, NACT regimen (anthracycline/taxane-based or other), pathological response at surgery, and recurrence patterns.

Pathological complete response was defined as the absence of invasive tumor in both breast and lymph nodes (ypT0/Tis ypN0). Recurrences were classified as locoregional, distant non-CNS (e.g., bone, lung, liver), or CNS (brain parenchyma or leptomeningeal disease). Data regarding the initial recurrence was extracted and presented. Event-free survival (EFS) was defined as the time between NACT initiation and recurrence, progressive disease or death.

Statistical analyses were performed using R version 4.4.0 (2024-04-04). Categorical variables were compared with Chi-square test. Survival was estimated with the Kaplan-Meier method and compared with the log-rank test. A two-sided p-value <0.05 was considered statistically significant.

## 3 Results

The study cohort comprised 774 TNBC patients with a median age of 48 years (Interquartile range: 40–58). The majority presented with stage III disease (63.4 %), and most received anthracycline/taxane-based NACT (97 %). Due to the limited number of patients (n = 9) treated with platinum-based regimens, subgroup analysis based on this criterion was not conducted. Pathological complete response was achieved in 218 patients (28.2 %). Patients achieving pCR exhibited a lower incidence of stage III disease (52.8 % vs 67.6 %, p < 0.001), higher Ki67 expression (71.1 % vs 66.2 %, p = 0.006), and a greater prevalence of grade 3 disease (68.3 % vs 58.3 %). Among those who relapsed, there were no differences in treatment regimens between pCR and no pCR groups.

During a median follow-up of 61.6 months, 265 patients (34.2 %) experienced disease recurrence. The recurrence rate differed significantly between the pCR and non-pCR groups (10.1 % vs. 43.7 %, p < 0.01). The 5-year event-free survival (EFS) was also higher in the pCR group (87 % vs 47 %, p < 0.001).The incidence of CNS relapse was similar between both groups (5.0 % in pCR and 8.8 % in non-pCR, p = 0.11); however, among recurred patients, CNS involvement was more frequent in pCR group than non-pCR (50.0 % of relapses in pCR vs 20.2 % in non-pCR, p = 0.003). By contrast, extracranial distant recurrences and locorregional recurrences were more frequent among non-pCR patients (extracranial distant: 27.3 % vs. 60.1 %, p = 0.006; locoregional: 9.1 % vs. 37.9 %; p = 0.01). Contralateral breast recurrence was more frequent among pCR relapses (18.2 % vs. 2.1 %; p = 0.001).

## 4 Discussion

Our findings align with recent reports suggesting that TNBC patients who achieve pCR after NACT may continue with a disproportionately elevated risk of CNS recurrence. While patients achieving pCR demonstrated lower absolute rates of recurrence, among those who experienced disease progression, CNS involvement accounted for fully half of all recurrences in the pCR group compared to just 20 % in non-pCR patients. This pattern suggests that while pCR confers overall protection against recurrence, it may selectively fail to prevent CNS metastases in a subset of patients.

Several biological mechanisms could explain this observation. The blood-brain barrier likely creates a pharmacologic sanctuary, preventing adequate chemotherapy penetration and allowing residual tumor cells to persist in the CNS microenvironment [4]. Additionally, the very biological features that make tumors sensitive to systemic therapy – e.g. including specific molecular subtypes or DNA repair profiles - might coincidentally predispose to neurotropism [5]. Further investigation on mechanisms associated with treatment sensitivity and tumor recurrence are warranted. Our finding of increased contralateral breast cancer in pCR patients might be another evidence pointing towards the notion that treatment-responsive tumors may have distinct metastatic patterns.

On the other hand, the higher CNS relapse rate in pCR patients may reflect their prolonged survival, providing a longer window for CNS progression to manifest. This contrasts with non-pCR patients, whose earlier extracranial recurrences might preclude the detection of CNS disease.

The role of targeted therapies (e.g., PARP inhibitors, immune checkpoint inhibitors) in preventing CNS relapses also warrants investigation. The current data available suggests that immunotherapy containing regimens may not protect CNS adequately [1], as the incidence of CNS relapses remains similar in patients receiving the Keynote-522 regimen.

The study has several limitations that should be acknowledged. Its retrospective nature introduces potential biases in patient selection and follow-up. While our sample size is substantial, the absolute number of CNS events remains modest, limiting some subgroup analyses. 10.13039/100014337Furthermore, given that the institution is publicly funded, access to adjuvant treatments such as capecitabine and olaparib is restricted. Consequently, only a small proportion of patients received these medications, despite their established benefits in reducing the risk of recurrence and mortality.

These findings raise important questions for future research. Prospective studies should incorporate standardized CNS imaging protocols to better characterize the true incidence of asymptomatic brain metastases. There is also a need to investigate the molecular characteristics of tumors from pCR patients who develop CNS recurrence, as these might reveal targetable pathways for prevention. Additionally, clinical trials should consider evaluating CNS-specific adjuvant therapies for high-risk pCR patients.

## 5 Conclusion

This retrospective cohort study demonstrates that while TNBC patients achieving pCR after NACT have reduced overall recurrence risk, they remain vulnerable to CNS relapse. Most notably, among pCR patients who experience disease progression, CNS metastases predominate, representing most recurrence events. This distinct pattern of failure highlights the need for modified surveillance strategies that specifically address CNS risk in pCR patients, as well as the development of targeted interventions to prevent neurotropic progression.

## CRediT authorship contribution statement

**Gabriel Berlingieri Polho:** Writing – review & editing, Writing – original draft, Methodology, Investigation, Formal analysis, Data curation, Conceptualization. **Yumi Ricucci Shinkado:** Writing – original draft, Methodology, Investigation, Data curation, Conceptualization. **Leticia Kimie Murazawa:** Writing – review & editing, Methodology, Investigation, Formal analysis, Data curation. **Vinicius Vitor Oliveira:** Writing – review & editing, Methodology, Investigation, Data curation. **Victor Rocha Pinheiro:** Writing – review & editing, Methodology, Investigation, Data curation. **Diana del Cisne Pineda Labanda:** Writing – review & editing, Methodology, Investigation, Data curation. **Romualdo Barroso-Sousa:** Writing – review & editing, Visualization, Validation, Supervision, Project administration, Investigation, Conceptualization. **Luciana Rodrigues Carvalho Barros:** Writing – review & editing, Visualization, Resources, Methodology, Investigation, Data curation. **Laura Testa:** Writing – review & editing, Visualization, Project administration, Methodology, Investigation, Conceptualization. **Renata Colombo Bonadio:** Writing – original draft, Validation, Supervision, Project administration, Methodology, Formal analysis, Data curation, Conceptualization.

## Declaration of competing interest

The authors declare the following financial interests/personal relationships which may be considered as potential competing interests: Gabriel Berlingieri Polho reports travel was provided by Libbs Pharmaceutical. Renata Colombo Bonadio reports a relationship with Daiichi Sankyo Inc that includes: consulting or advisory, speaking and lecture fees, and travel reimbursement. Renata Colombo Bonadio reports a relationship with Adium Pharma SA that includes: consulting or advisory and speaking and lecture fees. Renata Colombo Bonadio reports a relationship with Gilead Sciences Inc that includes: consulting or advisory and speaking and lecture fees. Renata Colombo Bonadio reports a relationship with Merck Sharp & Dohme UK Ltd that includes: consulting or advisory and speaking and lecture fees. Renata Colombo Bonadio reports a relationship with Bristol-Myers Squibb Company that includes: consulting or advisory and speaking and lecture fees. Renata Colombo Bonadio reports a relationship with AstraZeneca Pharmaceuticals LP that includes: consulting or advisory, funding grants, and speaking and lecture fees. Renata Colombo Bonadio reports a relationship with Eli Lilly and Company that includes: speaking and lecture fees. Renata Colombo Bonadio reports a relationship with Pfizer that includes: consulting or advisory and speaking and lecture fees. Renata Colombo Bonadio reports a relationship with Ache Pharmaceutical Laboratories SA that includes: consulting or advisory and speaking and lecture fees. Renata Colombo Bonadio reports a relationship with Novartis that includes: funding grants. Laura Testa reports a relationship with Daiichi Sankyo Inc that includes: consulting or advisory and speaking and lecture fees. Laura Testa reports a relationship with Merck & Co Inc that includes: consulting or advisory and speaking and lecture fees. Laura Testa reports a relationship with AstraZeneca Pharmaceuticals LP that includes: consulting or advisory and speaking and lecture fees. Laura Testa reports a relationship with Pfizer that includes: consulting or advisory and speaking and lecture fees. Laura Testa reports a relationship with Eli Lilly and Company that includes: consulting or advisory and speaking and lecture fees. Laura Testa reports a relationship with Novartis that includes: consulting or advisory, funding grants, and speaking and lecture fees. Laura Testa reports a relationship with Roche Pharma AG that includes: speaking and lecture fees. Laura Testa reports a relationship with Gilead Sciences Inc that includes: speaking and lecture fees. Romualdo Barroso-Sousa reports a relationship with AstraZeneca Pharmaceuticals LP that includes: consulting or advisory, funding grants, and speaking and lecture fees. Romualdo Barroso-Sousa reports a relationship with Daiichi Sankyo Inc that includes: consulting or advisory, funding grants, and speaking and lecture fees. Romualdo Barroso-Sousa reports a relationship with Eli Lilly and Company that includes: consulting or advisory and speaking and lecture fees. Romualdo Barroso-Sousa reports a relationship with Gilead Sciences Inc that includes: consulting or advisory and speaking and lecture fees. Romualdo Barroso-Sousa reports a relationship with Libbs Pharmaceutical that includes: consulting or advisory and speaking and lecture fees. Romualdo Barroso-Sousa reports a relationship with Pfizer Inc that includes: consulting or advisory and speaking and lecture fees. Romualdo Barroso-Sousa reports a relationship with Novartis that includes: consulting or advisory and speaking and lecture fees. Romualdo Barroso-Sousa reports a relationship with Merck & Co Inc that includes: consulting or advisory and speaking and lecture fees. Romualdo Barroso-Sousa reports a relationship with Roche Pharma AG that includes: consulting or advisory and speaking and lecture fees. If there are other authors, they declare that they have no known competing financial interests or personal relationships that could have appeared to influence the work reported in this paper.
